# Improving RED algorithm congestion control by using the Markov decision process

**DOI:** 10.1038/s41598-022-17528-x

**Published:** 2022-08-03

**Authors:** Amar A. Mahawish, Hassan J. Hassan

**Affiliations:** 1 Computer Engineering Department, University of Technology-Iraq, Baghdad, Iraq; 2 Computer Engineering Department, Al-Iraqia University, Baghdad, Iraq

**Keywords:** Engineering, Electrical and electronic engineering

## Abstract

Congestion control plays an essential role on the internet to manage overload, which affects data transmission performance. The random early detection (RED) algorithm belongs to active queue management (AQM), which is used to manage internet traffic. The RED is used to eliminate weakness in default control of the Transport Control Protocol (TCP) drop-tail mechanism. The drawback of RED is parameter tuning, while adaptive RED (ARED) automatically adjusts these parameters. In this study, the suggested algorithm, the Markov decision process RED (MDPRED) uses the Markov decision process (MDP) to suitably adapt values for queue weight in the RED algorithm based on average queue length to enhance the performance of the traditional RED during TCP Slow Startup phase. This study is conducted based on fluctuations among the rate of service, queuing weight, and the mean queue length by using open-source network simulator NS3. The study shows efficient results by fluctuating end-to-end packet throughput and fast response to the inception of congestion in the network. The modified algorithm achieves a low level of drop packets by evaluating the results with other five algorithms, which is done by increasing the algorithm’s response when the average queue size becomes close to the maximum queue length threshold.

## Introduction

The high-speed network and rapid growth of user numbers make congestion control more attractive for researchers. The congestion control algorithm increases the Quality of Service (QoS) by managing the overload in the router queue, increasing the throughput, and reducing the drop of packets in a network. The router in the network has a limited buffer, while congestion makes the buffer full, causing packets to drop. In TCP protocol the congestion effect by two main phases the Slow Startup phase which is responsible on seeking the capacity of path between two end points by increasing the rate exponentially, and then the Congestion Avoidance phase takes place to manage the sending rate with linear increasing rate. However, the default active queue management (AQM)^1^ algorithm is used as congestion control in Congestion Avoidance phase to reduce packet dropping and maintain the stability of data flow in the router’s queue. Many queue management algorithms have been developed to handle congestion that focused on managing congestion in Congestion Avoidance phase, such as the Random Early Detection (RED) algorithm^2^, Adaptive Random Early Detection (ARED) algorithm^3^, Gentle Random Early Detection (GRED)^4^, NonLinear Random Early Detection (NLRED)^5^, and Reconfigurable NonLinear Gentle Random Early Detection (RNLGRED)^6^.

The RED algorithm is used to overcome the drawback of the drop-tail (DT) mechanism used in Transmission Control Protocol/Internet Protocol (TCP/IP). While the ARED is developed to overcome the drawback of RED by dynamically updating the value of $max_P$ to keep the avg closer to the target queue size. The target queue size is selected halfway of the two thresholds, $min_{th}$ and $max_{th}$. By comparing the avg and target queue size, $max_P$ can be updated to minimize the gap between the avg and the target queue. Nevertheless, the ARED fails when a large number of senders with a substantially varying data rate are sent. Also, both algorithms mange the congestion during the Congestion Avoidance not on the Slow Startup phase of TCP flow. The GRED algorithm used to increase dropping probability slowly when the average queue exceeds $max_{th}$ by duplicate the $max_{th}$ to reduce the number of drop packets. The NLRED used exponential increasing probability unlike the above algorithms which depend on linear probability. The NLRED is useful in the traffic load that increasing exponentially while the RNLGRED used the Gentle feature (double $max_{th}$) and nonlinear increase of drop probability. The algorithms (GRED, NLRED and RNLGRED) have slow responses due to the static value of queue weight which has a problem when sender nodes used different data rates.

In internet today the short TCP flow is widely used such as web traffic objects and Internet of Things (IoT) applications. These type of traffic operate by alternate between active and inactive transmission periods, which mean that these traffics belongs to Slow Startup phase and default AQM algorithms have problems to manage such traffics. In this work the ON-OFF application used to reflect the Slow Startup flow rates.

To eliminate the problems in RED and ARED, the Markov Decision Process RED (MDPRED) algorithm has been suggested to give a fast response when many connections send different flow rates that belong to Slow Startup phase. The proposed algorithm used the feature of GRED by duplicate the $max_th$ in addition to dynamic adjust of queue weight. The MDP is a stochastic control process in discrete time; it has a set of states S and a set of actions A, and the MDP has a state transition matrix. The MDP is used to implement the reinforcement learning features, and it can be used with the RED algorithm to improve the performance by reducing the drop packets. The MDP needs to satisfy a Markov property that is independent of the past state, but it depends only on the present state.

The MDP has been used in many network fields as a stochastic process that builds an optimal model in uncertain environments to make proper action for a given state. For example^7^, used MDP to reduce the energy wastage in a wireless sensor network (WSN). Additionally, in^8^, the MDP was used to manage the network flow risks. In^9^, software-defined networking (SDN) used MDP as a defense strategy against distributed denial of service (DDoS) attacks. In^10^, the Wireless Body Area Network (WBAN), an optimal transmission policy between nodes was made based on the MDP to reduce energy consumption. In this study, the MDP is used to manage congestion in the router queue by taking proper action in a specific state to reduce drop packets. This paper examines the new algorithm with RED and ARED by using NS3 as a simulator to observe the number of packets dropped when the number of connections changes. “Related works” section describes the other work related to AQM and the Markov processes. “The proposed algorithm and model design” section introduces the suggested algorithm and the network design in this study. In “Simulation results and discussion” section, the implementation and analysis of the results are presented, while “Conclusion” section addresses the conclusion.

## Related works

Many algorithms have been developed to reduce the effect of congestion control in TCP/IP connections over the network^11^. The most recent AQM algorithms used optimization algorithm solutions to achieve optimal results. In^12^, the new model was used to control the window size of TCP packets with the help of the MDP to estimate the round-trip-time (RTT). That study used three types of congestion control states: CA-Open (Packets sent and acknowledgment received), CA-Recovered (the packets retransmitted), and CA-Loss (packets lost). The states of MDP are $s_0$ for the initial state, $s_1$ for the window size increasing state, $s_2$ for the congestion control state, and $s_3$ for the maximum window size. The proposed control shows the best performance in unreliable links and networks that have long been delayed by increasing or decreasing the window size based on congestion state. The problem with that study is in solving congestion with edge networks, while the most congestion issue is found in core networks, such as routers and switches.

The Markov-Modulated Bernoulli Arrival Process (MMBP)^13^ used in the AQM algorithm (such as Gentle Blue, which uses a dynamic calculation of $max_p$ based on queue length) is based on the Bernoulli Process (BP). In this method, the traffic properties have been correlated based on discrete time. The two states are used based on packet arrival probability, and in a specific slot time, the arrival packet to queue is evaluated to assign which state this packet’s rate belongs to. Study^7^ used an analysis based on the effect of varying the service rate within the queue in the system. The Poisson distribution is used to determine the servers’ distribution arrival rate when using variable service rates. The study focused on the management performance between the servers’ arrival rate and the service arrival rate, while improving the throughput and reducing the drop packet was ignored in that study.^14^ used an intelligent method to tune the AQM parameters depending on explicit congestion notification (ECN). In that study, there were two parts: congestion prediction and AQM parameter tuning. The first part used the neural network (NN), which learned from experience and ECN feedback, while the MDP was used to adjust the parameters. The study was used to improve the collision delay (CoDel), which depended on the delay of packets in the queue. The problem in that study was dependent on the ECN that started the congestion in the backward direction of transmission packets, which had some delay to handle congestion.

As mentioned before, the RED algorithm was the earliest algorithm that was developed based on the AQM principles. The RED algorithm needed to carefully tune its parameters; therefore, this algorithm struggled with the difficulty in adopting changes in the type of services and applications. ARED^3^ was designed to improve the weakness in RED by auto-tuning parameters using the automatic calculation of the drop probability. The drawback of the ARED algorithm was increasing complexity due to the auto tuning of the $max_p$.

The Gentle RED (GRED)^4^ was developed to improve the RED algorithm by duplicating the $max_th$ to twice $max_th$. This duplicate provided smooth tuning to the $max_P$. The problem with the GRED was the increased number of thresholds that needed to be selected carefully for a good result. Nonlinear RED (NLRED)^5^ by applying nonlinear change of drop probability, depending on the avg in the router queue. The researcher in^15^ incremented the queue weight when increasing the avg. The aggressive increase in the value of the queue weight enabled an increase in drop packets when the avg approached the double $max_th$. The problem in that study was also the increased number of parameters required to be carefully tuned.

All AQM algorithm mentioned above and the research done recently focused on congestion that occur in TCP Congestion Avoidance phase while the^16^ develop AQM that manage the congestion during TCP Slow Startup phase which is interested in this study.

## The proposed algorithm and model design

### The AQM algorithm

The default of AQM algorithm work is represent in RED algorithm. The RED Inception of the congestion depends on the average queue size (avg), two thresholds ($min_{th}$ and $max_{th}$) and maximum drop probability ($max_p$). Each packet arrives at the router queue; the algorithm calculates the avg by using exponential weight moving average (EWMA) as a low pass filter, which is shown in Eq. (1) as follows:

1 $$\begin{aligned} avg = \left( 1-w_q\right) avg+w_qq \end{aligned}$$

where $w_q$ is the queue weight and q is the current queue size. If the avg value is $min_{th}<avg<max_{th}$, the algorithm starts marking the arrival packets to the router’s queue using the Explicit Congestion Notification (ECN) available in TCP/IP. Therefore, to reduce the sending rate, the drop probability P can be calculated based on Eq. (2) as follows:

2 $$\begin{aligned} P= max_p(avg-min_{th})/(max_{th}+min_{th}) \end{aligned}$$

The RED algorithm starts dropping all incoming packets when avg exceeds $max_th$ to manage the congestion in the queue. The drawbacks of RED have a slow response to congestion and a difficulty tuning the parameter. Thus, these drawbacks make the algorithm work incorrectly when different applications and services use different data rates. The GRED has three values of thresholds ($min_th$,$max_th$ and double $max_th$) to reduce of drop probability slop curve. The proposed algorithm used three thresholds as in GRED and the dynamic value of $w_q$ selected based on the Markov process.

### Markov Process

The MDP depends on the combination of ($s_t$, $a_t$, $r_t$, t)^17^ with transitional probability to determine which action needs to be taken for a given state, which can be seen in Eq. (3) as follows:

3 $$\begin{aligned} P(s_{t+1}\vert s_t,a)=P(s_{t+1}=j\vert s_t=i,a=k) \end{aligned}$$

where $s_t$ and $s_{t+1}$ indicate the present state and the next state, respectively, while a indicates that an action needs to be taken. *i* and *j* can be 1,2,3, ... that represent states, where *j* is the next state and *i* is the current state; and k can be 1,2,3, ... to indicate which action is taken.

$r_t$ is the reward (return) from the environment to the agent after an action is applied to the current state, as shown in Eq. (4), and this reward can be the maximum or minimum. In this work, the reward represents a minimum number of packets dropped as follows:

4 $$\begin{aligned} R_t=\sum _{i=t}^{\infty } r_{i}=r_t+r_{t+1}+r_{t+2}+... \end{aligned}$$

The MDP includes the discount factor $\gamma$, which has a value between 0 and 1 to give a weight for future rewards. In this study, the value of $\gamma$=0 includes only the immediate reward without involving future rewards, which is shown in Eq. (5) as follows:

5 $$\begin{aligned} R_t=r_t+\sum _{i=t+1}^{\infty } \gamma ^i r_i \end{aligned}$$

To map MDP on the RED algorithm, there is a need to consider the average queue length, avg, as a state and the type of drop packets as an action. We assume four sets of states S=$s_1, s_2, s_3, s_4$. Then, we have the $4\times 4$ transition probability matrix shown in Eq. (6). $s_1$ means the avg state TCP for a slow start below a minimum threshold $(0< avg < min_{th})$, $s_2$ indicates the avg state between $min_{th}$ thresholds and halfway of the two thresholds $(min_{th}< avg < (min_{th} +max_{th})/2), s_3$ indicates the state when the avg is between halfway of the two thresholds and below the $max_{th} ((min_{th} + max_{th})/2< avg < max_{th})$, and $s_4$ indicates the state when the avg is greater than $max_{th}$ and less than double $max_{th}$ $(max_th<avg<2*max_th)$. We assume that there are three sets of action A=$a_1,a_2,a_3$, where $a_1$ represents the no-drop packet, while $a_2$ and $a_3$ indicate the unforced drop and the forced drop, respectively, as follows:

6 $$\begin{aligned} P_{ij} = \begin{bmatrix} P_{00} &{} P_{01} &{} P_{02} &{} P_{03} \\ P_{10} &{} P_{11} &{} P_{12} &{} P_{13} \\ P_{20} &{} P_{21} &{} P_{22} &{} P_{23} \\ P_{30} &{} P_{31} &{} P_{32} &{} P_{33} \\ \end{bmatrix} \end{aligned}$$

### Design and methodology

**Figure 1:**
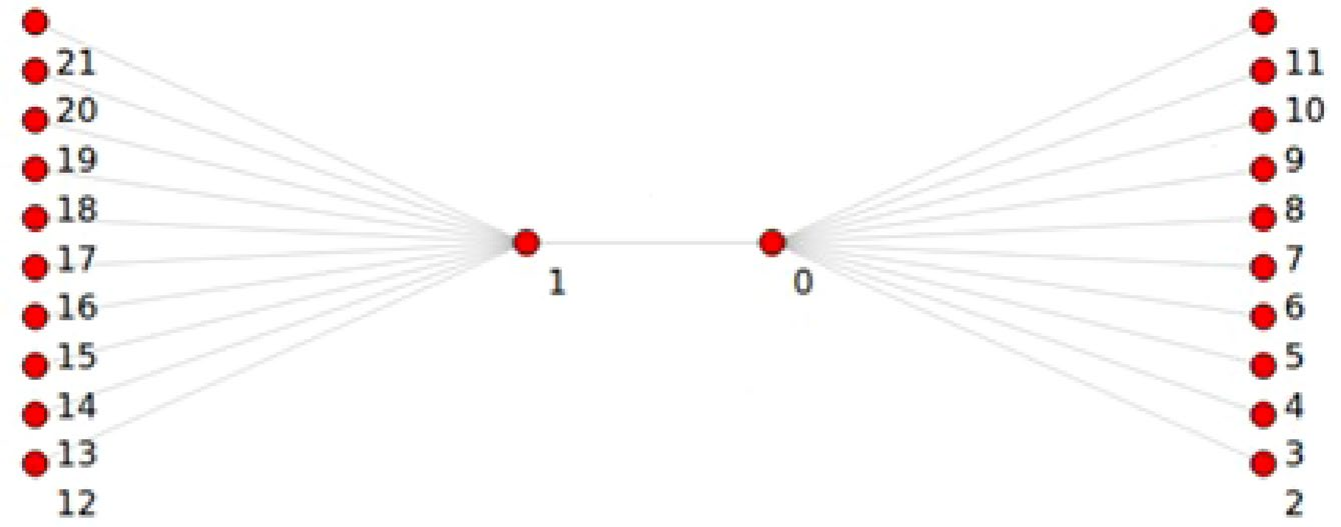
NS3 Point-To-Point Dumbbell topology before starting simulation.

**Figure 2:**
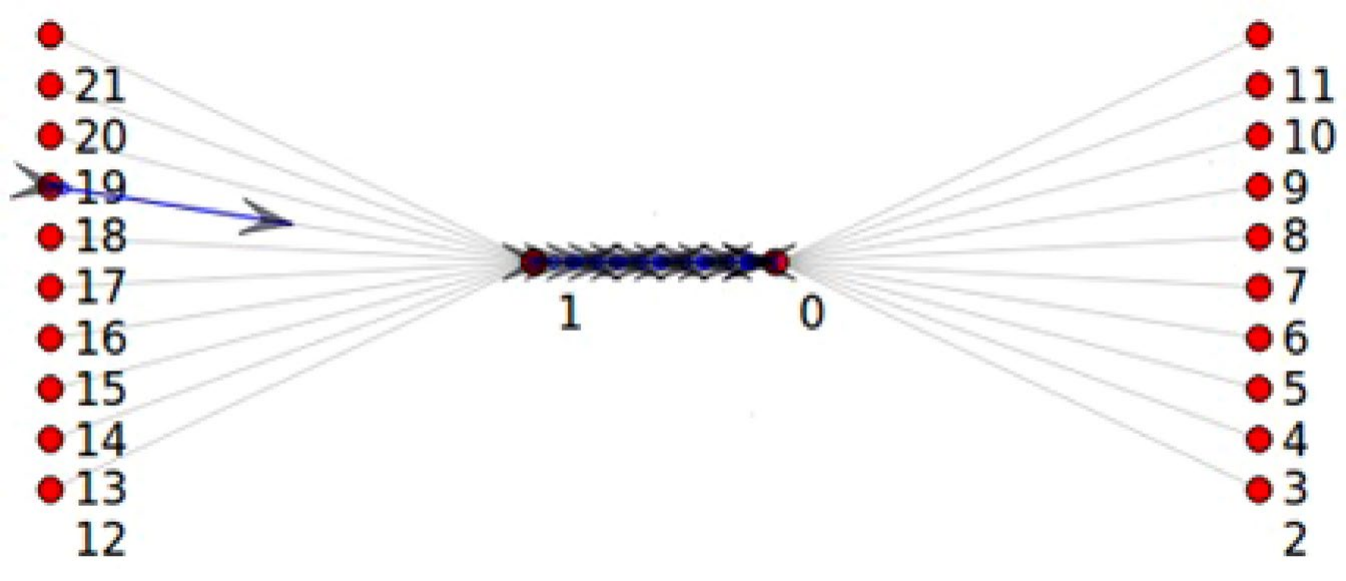
NS3 Point-To-Point Dumbbell topology after 10 sec of run time.

The Point-To-Point Dumbbell network topology built by NS3 with ON-OFF application was used in this study, as shown in Fig. 1. In the current study, the network has five nodes on each left and right side at beginning then increasing by 5 nodes in each simulation round up to 200 nodes, the source and the destination, respectively, and the two nodes in the middle that reflect the router in the core network create a bottleneck. Figure 1 shows that the sending nodes have not sent any packet, while in Fig. 2, the bottleneck link has been congested, and the drop packets procedure started.

### TCP slow start

The TCP protocol has a default congestion control with four phases: slow start, congestion avoidance, fast retransmit, and fast recovery^18^. The slow start phase allows the TCP to inform the capacity of links in the transition path; this occurs by duplicating the window size per RTT of each acknowledgment received. If no acknowledgment is received, the TCP indicates that congestion occurs, and the congestion avoidance phase starts; then, the window size increases by one for all successful acknowledgments. Therefore, the TCP slow starts increasing exponentially in each RTT, which means that congestion can occur within a short time.

### Tuning algorithm parameters

**Figure 3:**
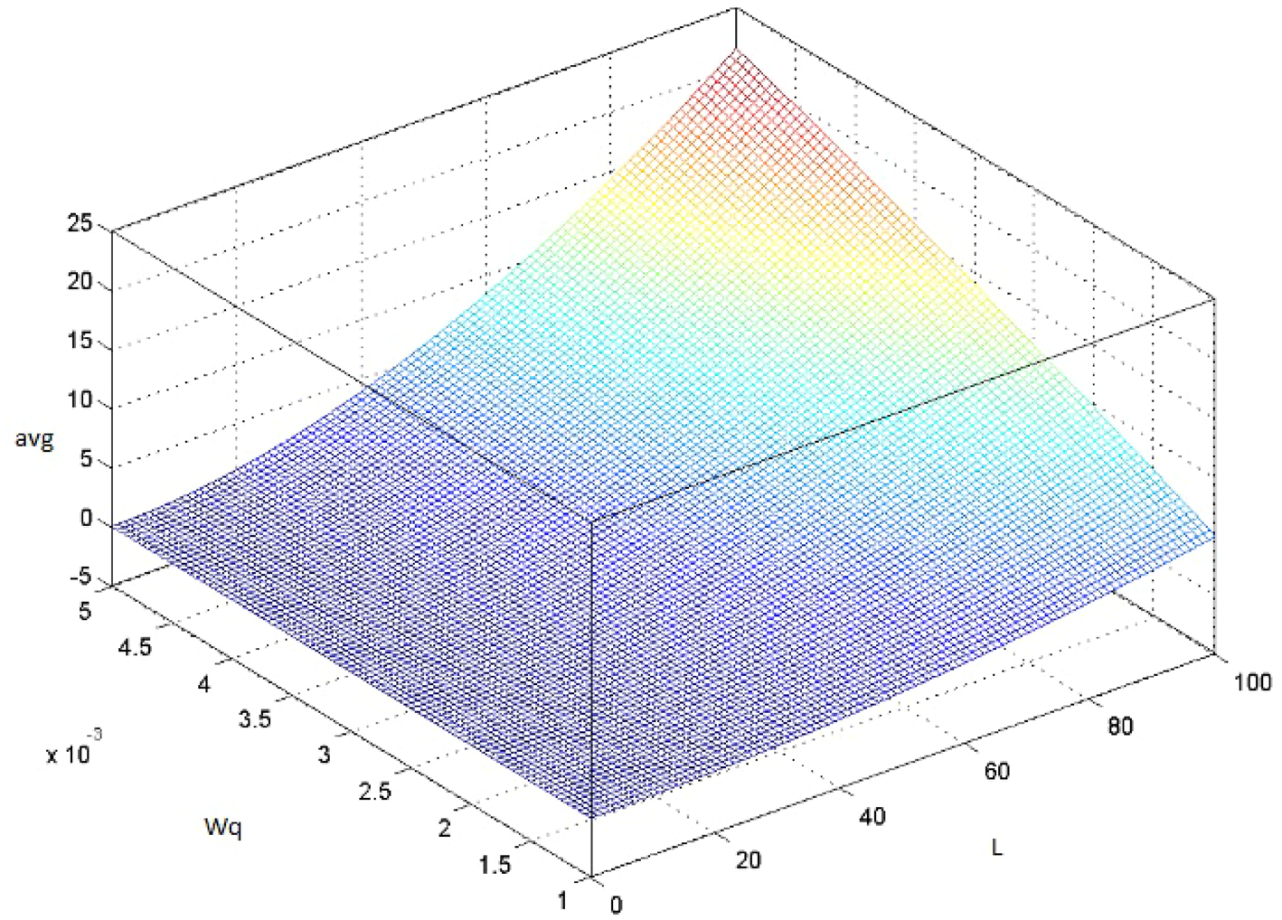
The effect of the avg as a function of $w_q$ and the load rate.

The default values in the RED algorithm parameters are $w_q$=0.002, $min_{th}$=5, $max_{th}$=15, $max_p$=1/50^2^. In the present study, the probability transition matrix $P_{ij}$ in Eq. (6) is calculated based on queue weight $w_q$. Equation (7) shows the relation between the load rate and $w_q$, as well as their effect on the avg value^2^ as follows:

7 $$\begin{aligned} avg=L+1+\frac{(1-w_q)^{(L+1)}-1}{w_q} \end{aligned}$$

where L represents the load rate of the sending packets. $w_q$ works as a time constant in a low pass filter on the average queue value, which reflects the response time and queue weight of incoming packets, as shown in Fig. 3. Therefore, if $w_q$ is too large, then the algorithm does not filter out the congestion that appeared in a short time, especially in the slow start of the TCP protocol, as mentioned in the previous subsection. If $w_q$ is too small, then the algorithm response to congestion is too slow to reflect the change in queue size, and the small value of the queue weight is suitable for the slow start phase in the TCP. In this study the initial value of $w_q$ set to zero and increment by 0.002 when the state of avg change to other state, these values selected to manage the exponential increase of TCP Slow Startup flow. Table 1 shows the range of the $w_q$ used in the suggested algorithm.

**Table 1:** The value of avg based on the queue length state.

State	Value of $w_q$
$0< avg < min_{th}$	0.002
$$min_{th}< avg < (min_{th}$$ + $$max_{th})$$/2	0.004
$(min_{th} + max_{th})/2< avg < max_{th}$	0.006
$max_{th}< avg < 2*max_{th}$	0.008

By using the values of the $w_q$, the MDP probability transition matrix is obtained, shown in Eq. (6) as follows:

8 $$\begin{aligned} P_{ij} = \begin{bmatrix} 0 &{} 0.002 &{} 0.002 &{} 0.002 \\ 0.004 &{} 0 &{} 0.004 &{} 0.004 \\ 0.006 &{} 0.006 &{} 0 &{} 0.006 \\ 0.008 &{} 0.008 &{} 0.008 &{} 0 \\ \end{bmatrix} \end{aligned}$$

The diagonal matrix assigns a zero value, which means there is no change in the queue weight value if the state is the same. The parameters of the implemented network are shown in Table 2:

**Table 2:** The parameter values of the implemented algorithm.

Parameter name	Value
Packet size	512 Bytes
Data rate	10 Mbps
Bottleneck link capacity	1 Mbps
$min_{th}$	5
$max_{th}$	15


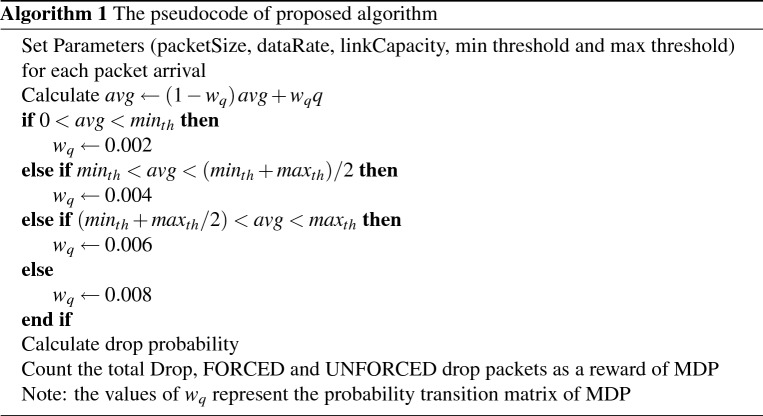


## Simulation results and Discussion

The NS3 simulation is used to analyze and evaluate the new algorithm, MDPRED, against five algorithms: RED, ARED, GRED, NLRED and RNLGRED based on the total drop, forced drop, and unforced drop packets. In this paper, the performance of the suggested algorithm has been analyzed based on varying of the sending rates and the effect on the average queue length^19^ by increasing the number of nodes in the Point-To-Point Dumbbell network topology.

**Figure 4:**
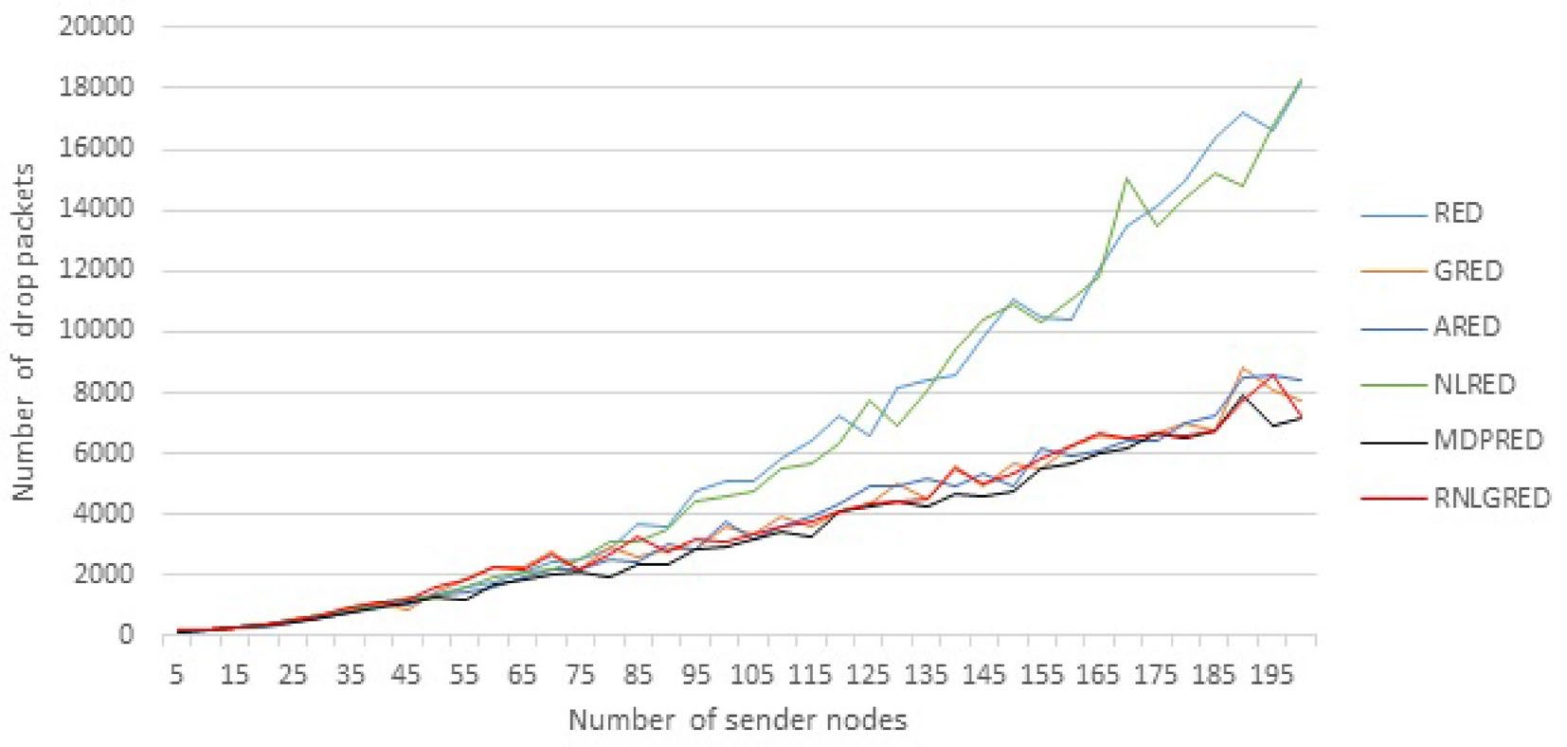
Drop Packets based on the different data loads.

In Table 3, which shows samples of implementation results for six algorithms for five different node readings, the total number of dropped packets is displayed. The MDPRED provides the optimum performance, especially as data load increases, according to the table. In each simulation run from 5 to 200 nodes, Fig. 4 displays the six algorithms dropping packets when the number of sending nodes is increased by 5 (Increasing nodes means increasing data load on a network). The figure shows that when the data rates increase, the dropping gap between the MDPRED and the two algorithms (RED and NLRED) increases. While the dropping rates of the other three algorithms are comparable to the proposed algorithm, Table 3 and Fig. 4 demonstrate that the MDPRED has the lowest dropping rates of all the algorithms. Accordingly , the MDPRED performs better and returns less dropped packets.

**Table 3:** The sample of implementation reading for number of drop packets.

No. of Nodes	RED	ARED	GRED	NLRED	RNLGRED	MDPRED
5	162	140	174	161	174	142
50	1348	1320	1433	1315	1575	1241
100	5116	3731	3562	4579	3094	2940
150	11029	4905	5705	10877	5361	4725
200	18196	8387	7762	18300	7251	7180

**Figure 5:**
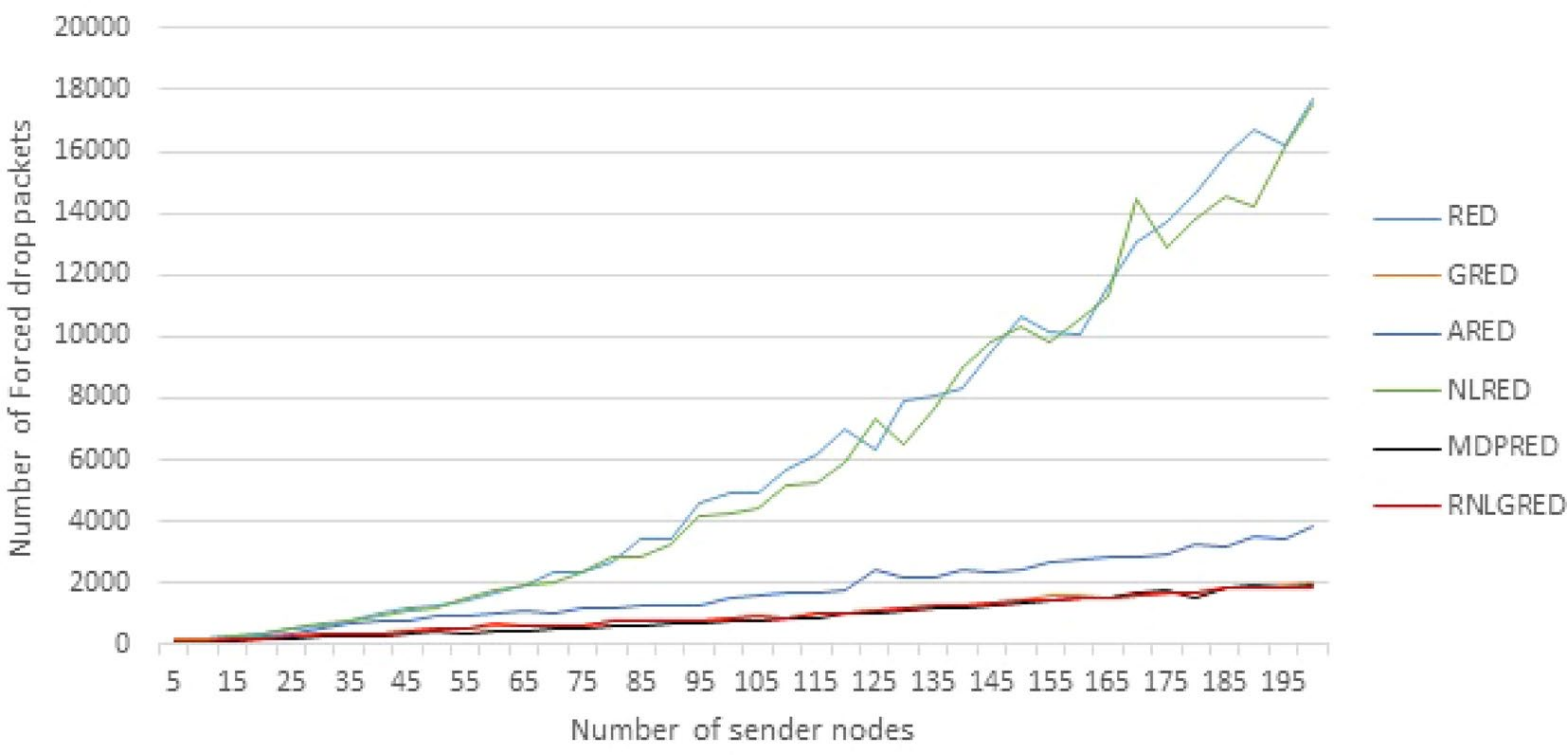
The force packets drop based on the different data loads.

The congestion algorithm can either drop the packets before enqueueing them in the router queue as forced drop or mark the incoming packets with congestion notification of $max_p$ to be dropped later when congestion increases as unforced drop. Similar to Tables 3 and 4 presents some of implementation readings for Forced drop packets, while Fig. 5 presents all readings. Moreover Table 4 and Fig. 5 show that the MDPRED mostly has less forced drop than the other five algorithms, which means that the new algorithm has better performance than the default RED, ARED and NLRED algorithms. While the forced drop rate of GRED and RNLGRED algorithms is close to MDPRED, but still the new algorithm has less Forced drop packets, so the new algorithm is less aggressive to deal with congestion by reducing forced drop.

**Table 4:** The sample of implementation reading for number of Forced drop packets.

No. of Nodes	RED	ARED	GRED	NLRED	RNLGRED	MDPRED
5	160	129	117	160	117	79
50	1262	911	496	1201	496	469
100	4899	1547	848	4281	848	772
150	10679	2474	1421	10348	1419	1340
200	17748	3869	1979	17571	1859	1975

**Figure 6:**
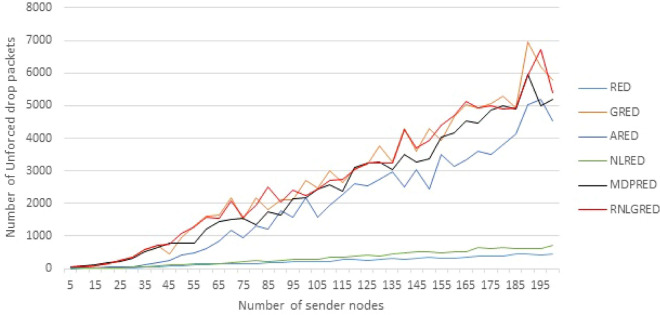
The unforced Packets drop based on the different data loads.

Table 5 presents some of implementation readings for Unforced drop packets, while all readings of unforced drop packets are shown in Fig. 6. The unforced drop packets (marked packets) in the MDPRED present better performance than those in the other three algorithms (RED, ARED, and NLRED). On the other hand, the GRED and RNLGRED provide the best results, but still the MDPRED algorithm gives the best result in overall drop packets as shown in Fig. 4 and Table 3. According to Fig. 6, it can be concluded that the new algorithm gives an acceptable rate in Unforced dropping packets, which means giving a chance to deliver the packets to the destination without dropping if the congestion in the network reduces.

**Table 5:** The sample of implementation reading for number of Unforced drop packets.

No. of Nodes	RED	ARED	GRED	NLRED	RNLGRED	MDPRED
5	2	11	57	1	57	63
50	86	409	937	114	1079	772
100	217	2184	2714	298	2246	2168
150	350	2431	4284	529	3942	3385
200	448	4518	5783	729	5392	5205

## Conclusion

The MDPRED is tuning the time response of the queue to the congestion by adopting the queue weight $w_q$, which works as a timely response in a low pass filter. The newly designed algorithm depends on using the Markov process by setting the states and actions. The MDPRED has a better minimum drop packet (as a reward) than the RED, ARED, GRED, NLRED and RNLGRED algorithms. The performance of MDPRED algorithm was tested when increasing the traffic load by increasing the number of nodes connected in the network from 5 to 200 nodes. The MDPRED has a less percentage of drop packets than the other five algorithms, which means that the suggested algorithm has higher performance to deal with the drop packets.

## Data Availability

The paper entitled “Improving RED algorithm congestion control by using the Markov Decision Process” has dataset collected from following hyperlinks: 1. The simulation and result of paper obtained by using NS3 “Network simulator 3” https://www.nsnam.org/ 2. In https://www.nsnam.org/doxygen/red-queue-disc_8cc_source.html all five compared algorithms result (dataset) available. For example, the line 95 in hyperlink above the Gentle can be either true or false to reflect RED and GRED algorithm respectively, while line code 100 the ARED can be implement by set it to true and the code line 115 represent the NLRED set to true with Gentle set to false or true to implement the NLRED or RNLGRED respectively.
