# Author Correction: Improving RED algorithm congestion control by using the Markov decision process

**DOI:** 10.1038/s41598-022-23714-8

**Published:** 2022-11-16

**Authors:** AmarA. Mahawish, Hassan J. Hassan

**Affiliations:** 1 Computer Engineering Department, University of Technology-Iraq, Baghdad, Iraq; 2 Computer Engineering Department, Al-Iraqia University, Baghdad, Iraq

Correction to: *Scientific Reports* 10.1038/s41598-022-17528-x, published online 03 August 2022

The original version of this Article contained an error, where “max_th_” was incorrectly given as “maxth”.

As a result, in the Introduction,

“The RED algorithm is used to overcome the drawback of the drop-tail (DT) mechanism used in Transmission Control Protocol/Internet Protocol (TCP/IP). While the ARED is developed to overcome the drawback of RED by dynamically updating the value of *max*_*P*_ to keep the avg closer to the target queue size. The target queue size is selected halfway of the two thresholds, *min*_*t*_*h* and *max*_*t*_*h*. By comparing the avg and target queue size, *max*_*P*_ can be updated to minimize the gap between the avg and the target queue. Nevertheless, the ARED fails when a large number of senders with a substantially varying data rate are sent. Also, both algorithms mange the congestion during the Congestion Avoidance not on the Slow Startup phase of TCP flow. The GRED algorithm used to increase dropping probability slowly when the average queue exceeds *max*_*t*_*h* by duplicate the *max*_*t*_*h* to reduce the number of drop packets. The NLRED used exponential increasing probability unlike the above algorithms which depend on linear probability. The NLRED is useful in the traffic load that increasing exponentially while the RNLGRED used the Gentle feature (double *max*_*t*_*h*) and nonlinear increase of drop probability. The algorithms (GRED, NLRED and RNLGRED) have slow responses due to the static value of queue weight which has a problem when sender nodes used different data rates.”

now reads:

“The RED algorithm is used to overcome the drawback of the drop-tail (DT) mechanism used in Transmission Control Protocol/Internet Protocol (TCP/IP). While the ARED is developed to overcome the drawback of RED by dynamically updating the value of *max*_*P*_ to keep the avg closer to the target queue size. The target queue size is selected halfway of the two thresholds, *min*_*th*_ and *max*_*th*_. By comparing the avg and target queue size, *max*_*P*_ can be updated to minimize the gap between the avg and the target queue. Nevertheless, the ARED fails when a large number of senders with a substantially varying data rate are sent. Also, both algorithms mange the congestion during the Congestion Avoidance not on the Slow Startup phase of TCP flow. The GRED algorithm used to increase dropping probability slowly when the average queue exceeds *max*_*th*_ by duplicate the *max*_*th*_ to reduce the number of drop packets. The NLRED used exponential increasing probability unlike the above algorithms which depend on linear probability. The NLRED is useful in the traffic load that increasing exponentially while the RNLGRED used the Gentle feature (double *max*_*th*_) and nonlinear increase of drop probability. The algorithms (GRED, NLRED and RNLGRED) have slow responses due to the static value of queue weight which has a problem when sender nodes used different data rates.”

In addition, Table 2 contained an error,

**Table 2:** The parameter values of the implemented algorithm

Parameter name	Value
0 < *avg* < *min*_*th*_	0.001
Packet size	512 Bytes
Data rate	10 Mbps
Bottleneck link capacity	1 Mbps
*min*_*th*_	5
*max*_*th*_	15

now reads:

**Table 2:** The parameter values of the implemented algorithm

Parameter name	Value
Packet size	512 Bytes
Data rate	10 Mbps
Bottleneck link capacity	1 Mbps
*min*_*th*_	5
*max*_*th*_	15

The original Article has been corrected.

